# Dietary composition of adult eosinophilic esophagitis patients is related to disease severity

**DOI:** 10.1002/iid3.1206

**Published:** 2024-03-08

**Authors:** Simone R. B. M. Eussen, Sanne Wielders, Willemijn E. de Rooij, Marleen T. J. Van Ampting, Betty C. A. M. Van Esch, Jeanne H. M. de Vries, Albert J. Bredenoord, Berber Vlieg‐Boerstra

**Affiliations:** ^1^ Danone Nutricia Research Utrecht The Netherlands; ^2^ Division of Human Nutrition Wageningen University Wageningen The Netherlands; ^3^ Department of Gastroenterology & Hepatology Amsterdam University Medical Center Amsterdam The Netherlands; ^4^ Division of Pharmacology, Utrecht Institute for Pharmaceutical Sciences, Faculty of Science Utrecht University Utrecht The Netherlands; ^5^ Department of Pediatrics OLVG Hospital Amsterdam The Netherlands

**Keywords:** anti‐inflammatory diet, disease severity, eosinophilic esophagitis, foods, immunomodulation, inflammation, nutrients

## Abstract

**Background:**

In addition to the elimination diet, dietary composition may influence disease severity in patients with eosinophilic esophagitis (EoE) through modulation of the immune response.

**Aim:**

To explore the immunomodulatory role of nutrition before and during elimination diet in adult EoE patients.

**Methods:**

Nutritional intake was assessed in 39 Dutch adult EoE patients participating in the Supplemental Elemental Trial (Dutch trial registry NL6014, NTR6778) using 3‐day food diaries. In this randomized controlled trial, diagnosed patients received either a four‐food elimination diet alone (FFED) or FFED with addition of an amino acid‐based formula for 6 weeks. Multiple linear regression analyses were performed to assess associations between the intake of nutrients and food groups per 1000 kCal and peak eosinophil count/high power field (PEC), both at baseline and after 6 weeks.

**Results:**

At baseline, we found a statistically significant negative (thus favorable) relationship between the intake of protein, total fat, phosphorus, zinc, vitamin B12, folate, and milk products and PEC (*p* < .05), while calcium (*p* = .058) and full‐fat cheese/curd (*p* = .056) were borderline (favorably) significant. In contrast, total carbohydrates, prepacked fruit juice, and white bread were significantly positively (unfavorable) related to PEC (*p* < .05), while ultra‐processed meals (*p* = .059) were borderline (unfavorably) significant. After dietary intervention, coffee/tea were significantly negatively (favorably) related to PEC, hummus/legumes were significantly positively (unfavorably) related with PEC, while peanuts were borderline significantly positively related (*p* = .058).

**Conclusion:**

Dietary composition may be related to inflammation in adult EoE patients. High‐quality and anti‐inflammatory diets may be a promising adjuvant therapy in the dietary management of EoE.

## 1 INTRODUCTION

Eosinophilic esophagitis (EoE) is a chronic immune‐mediated disease characterized by inflammation of the esophagus, food impaction, and dysphagia.^1^, ^2^ During the last two decades, the incidence and prevalence of EoE in adults have increased significantly, especially in the Western countries.^3^, ^4^, ^5^ Currently, worldwide, an estimate of 50–100 cases/100,000 citizens are diagnosed with this allergic disease.^4^ To date, there is no explanation for the rapid increase of prevalence of EoE over the past two decades.

Diagnosis of EoE is confirmed if an esophageal biopsy contains at least 15 eosinophils per “High Power Field” (HPF),^6^, ^7^, ^8^, ^9^ indicating esophageal inflammation. Although the disease pathology of EoE is complex, food allergens seem to have a causative role. The avoidance diet is one of the major therapeutic options in EoE.^1^, ^2^ Typically, two‐food elimination diets (dairy and gluten/wheat), four‐food elimination diets (FFED) (cow's milk, gluten/wheat, eggs, and soy or legumes), or six‐food elimination diets (cow's milk, gluten/wheat, eggs, soy, peanuts/nuts, and fish/shellfish) are applied.^6^, ^7^, ^9^, ^10^, ^11^, ^12^ Alternatively, amino acid‐based diets, that is, liquid formulas based on amino acids, are most effective in achieving disease remission. However, poor taste and lack of solid food intake make adherence to elemental diets challenging.^8^, ^9^, ^12^, ^13^

As EoE is a chronic disease, symptoms and inflammation recur if management is discontinued. It has been shown that the exclusion of food allergens from the diet induces histological and clinical remission in patients, while the introduction of food allergens after remission leads to rapid disease relapse.^11^ Thus, EoE patients who achieve histologic remission are subject to long‐term maintenance dietary elimination therapy. Consequently, the impact on quality of life is significant due to the required efforts to adhere to dietary constraints, limited food choice and the psycho‐sociological consequences. Hence, long‐term adherence to maintenance diet therapy is difficult and lower than expected.^14^ Therefore, adjuvant management options to support and alleviate dietary maintenance therapy are urgently needed.

Ample evidence has accumulated in recent years for the immunomodulatory effects of foods and nutrients.^15^, ^16^ The European Food Safety Authority has recognized the immunomodulatory effects of the following nutrients: proteins, iron, selenium, copper, zinc, and vitamins (A, B6, B12, C, D, folate).^17^ Moreover, long‐chain polyunsaturated fatty acids (LCPUFAs) are known for their immune‐modulatory properties. These fatty acids serve as specific precursors of immune regulatory eicosanoids.^18^ Additionally, dietary components, for instance, vitamin A, iron, dietary fibers, and zinc, have shown to have a local effect on both the mucosal integrity, as well as on the mucosal cells of the gastrointestinal tract.^19^, ^20^, ^21^, ^22^, ^23^, ^24^, ^25^, ^26^, ^27^ Furthermore, nutritional intake of plant‐based and animal‐based proteins, fruits and vegetables, dietary fibers, fat, LCPUFAs, and polyphenols influence the gut microbiota composition,^28^, ^29^, ^30^, ^31^, ^32^ which may indirectly influence the inflammatory response. Finally, due to epigenetic effects, nutrients may alter gene expression together with disease susceptibility.^33^

We hypothesized that through immunomodulatory effects, the nutritional composition of the diet (other than elimination) may play an important role in the pathology of EoE. If that is true, a healthful high‐quality diet with anti‐inflammatory properties could be applied as adjuvant therapy to support management and disease control during the maintenance phase of EoE.

Studies investigating EoE patients' habitual dietary intake and its link to disease severity are scarce. To the best of our knowledge, only one previous study by our group examined the relationship between the habitual diet and esophagus inflammation in a small adult EoE study population, showing promising results.^34^ Results indicated a statistically significant negative (and thus favorable) relationship between the intake of dietary fiber, soy, rice/pasta and iron, and eosinophil count, whereas the intake of phosphorus was significantly positively (and thus unfavorably) associated to eosinophil count. In addition, we showed that the composition of the habitual diet of adult EoE patients had several proinflammatory and thus unfavorable immunomodulatory properties, just as the general Dutch population, however, EoE patients had lower overall diet quality scores than the general population.^35^

Based on these results, we performed this exploratory study aimed to evaluate associations between the nutritional intake and disease severity in another adult patient population to gain more insights into the possible aggregating and protecting effects of nutrients and food groups involved in the pathophysiology of EoE.

## 2 METHODS

### 2.1 Study design and study population

Nutritional intake was assessed in 39 out of 40 Dutch adult EoE patients participating in the single‐center, open‐label, randomized controlled Supplemental Elemental Trial (Dutch trial registry NL6014, NTR6778) between December 2017 and March 2020. Adult patients were eligible for enrollment if EoE was diagnosed according to the consensus guidelines, defined as having symptoms of esophageal dysfunction (Straumann Dysphagia Instrument score of ≥1) and ≥15 eosinophils per microscopic HPF on baseline biopsy.^1^ Exclusion criteria included severe comorbidities, the use of systemic corticosteroids, leukotriene inhibitors, monoclonal antibodies, or anticoagulants in the month preceding the study, and the inability to stop the use of topical corticosteroids. Further details of inclusion and exclusion criteria can be found elsewhere.^36^ After confirming patients' eligibility and obtaining written informed consent for study participation, the patients were randomized to receive either a standard FFED, excluding cow's milk, eggs, soy, and gluten, or FFED with the addition of an amino acid‐based formula (Neocate Junior) (FFED + AAF) providing 30% of the calory needs for 6 weeks.^36^ At baseline and after 6 weeks of dietary intervention, histological disease activity (peak eosinophil count/HPF (PEC)) was measured by histological examination and dietary intake was assessed. The study was approved by the Amsterdam University Medical Center Institutional Review Board.

### 2.2 Outcome parameters

#### 2.2.1 Histological disease activity

An esophagogastroduodenoscopy (EGD) was performed at baseline and after 6 weeks of dietary intervention to assess PEC.^36^ All endoscopic images were scored according to the Endoscopic Reference Score by a single‐blinded gastroenterologist with expertise in EoE.^36^, ^37^

#### 2.2.2 Nutritional intake assessment

At baseline and after 6 weeks of dietary intervention, patients' habitual diet and nutritional intake during FFED or FFED + AAF were assessed using a 3‐day food diary. In addition, in order not to miss any information on infrequently consumed nutrients and foods, patients' intake of docosahexaenoic acid, eicosapentaenoic acid, and fish was assessed by means of a Food Frequency Questionnaire.^38^ Patients were asked to record amounts of foods used in grams or household portion sizes, as well as to record types and brand names of foods consumed in detail. Portion sizes were coded using Compl‐eat software (Wageningen University, Human Nutrition WUR).^39^ Patients also recorded the type and use of dietary supplements (yes/no/infrequently). The food diaries were reviewed by an allergy specialist dietitian with experience in EoE using a checklist for completeness. Patients were contacted in case of unclear or missing data. This was done promptly to minimize the length of the recall period and reduce the risk of recall bias. The average nutrient intake of the 3‐day diaries (without supplements) was calculated using the Dutch NEVO‐online Food Composition Database^40^ and Compl‐eat software^39^ for energy (kCal) and 41 nutrients. All foods consumed were allocated to one of the 23 main food groups, 83 subgroups at level 1 or 19 subgroups at level 2 adapted from Compl‐eat (Supporting Information S2: Table 1).

#### 2.2.3 Dietary advice

All patients received dietary advice from the dietitian regarding the FFED or FFED + AAF. The dietitian recommended that the diet met the Dutch dietary guidelines as set by the Dutch National Health Council^41^ with adequate replacement of cow's milk by AAF or plant‐based milk replacers supplemented with calcium, vitamin B2, and D. Information on individualized sample menus, brands, label reading, and recipes was provided to the patients. During the 6‐week intervention period, patients were monitored twice by the dietitian to evaluate diet compliance, adverse effects, and body weight maintenance.

#### 2.2.4 Statistical analyses

All nutritional intake data were calculated per 1000 kCal per day, to correct for differences between patients in energy intake. The Wilcoxon test was used to compare the intake of nutrients and food groups at baseline and the intake after the 6‐week intervention period. Subsequently, data were checked for model assumptions of normality, linearity, and homoscedasticity of residuals. In case of nonlinearity, a log‐transformation was used. Multivariate linear regression analyses were performed to investigate the relationships between the intake of nutrients and food groups per 1000 kCal and PEC, both at baseline and after 6 weeks. Gender and age were included as covariates into the multiple regression models. In addition, for the analyses at 6 weeks, baseline PEC and use of AAF (yes/no) were included as covariates. *p *< .05 were considered statistically significant. IBM SPSS Statistics version 20.0 (SPSS) was used for all statistical analyses.

## 3 RESULTS

### 3.1 Inclusion and patient characteristics

**Figure 1:**
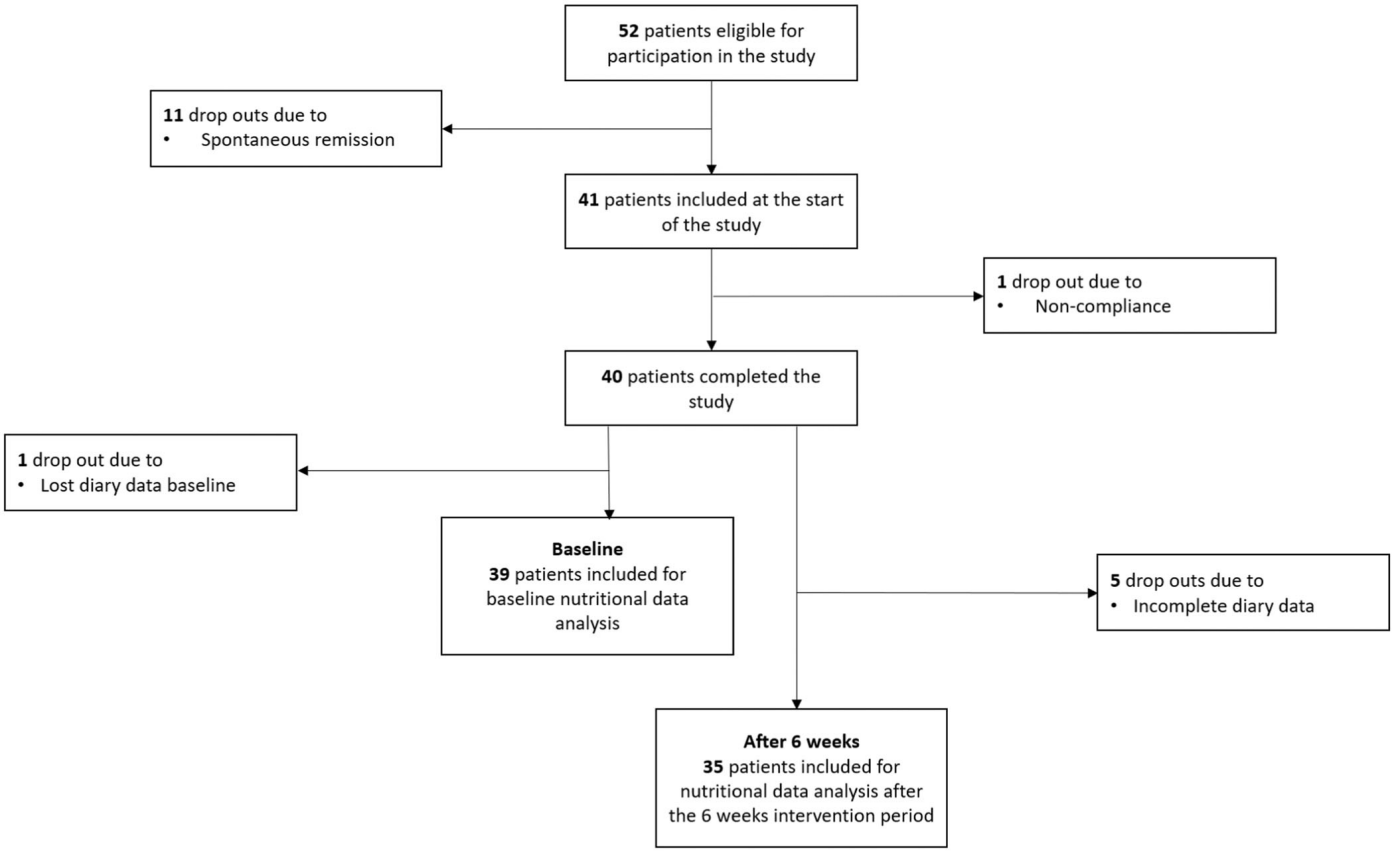
Flow chart of the patients included for data analysis.

At baseline, initially, 52 patients were enrolled and underwent EGD (Day 0), after which 11 patients were excluded due to spontaneous disease remission. Of the 41 patients who eventually were enrolled and participated in the study, one patient was excluded due to noncompliance to the diet during the 6‐week diet intervention period. Overall, of the 40 patients who completed the 6‐week diet intervention, 39 patients were eligible for dietary baseline analysis, while 35 patients were eligible after the 6‐week intervention period (Figure 1). The patient characteristics are depicted in Table 1. The median age was 34.2 years and 60% of the patients were males. Median body mass index (BMI) was 24.1 kg/m^2^ and 2.5%, 37.5%, and 5.0% of patients, were respectively, underweight (BMI < 18.5 kg/m^2^), overweight (25 ≤ BMI < 30 kg/m^2^), and obese (BMI ≥ 30 kg/m^2^), that is, comparable to the general Dutch population.^42^

**Table 1:** Baseline characteristics of all patients who completed the trial (*n* = 40) in both groups.

	FFED	FFED + AAF	*p* Value
	(*n* = 20)	(n = 20)	
Male gender, *n* (%)	12 (60)	12 (60)	ns
Age, *years*, median (IQR)	32.0 (27.5–43)	36.5 (29.25–42)	ns
Race, *Caucasian, n* (%)	19 (95)	19 (95)	ns
History of allergic disease, *n* (%)	17 (85)	17 (85)	ns
Allergic rhinitis	14 (70)	13 (65)	ns
Asthma	6 (30)	6 (30)	ns
Atopic dermatitis	5 (25)	7 (35)	ns
Food allergy	6 (30)	5 (25)	ns
Angioedema	1 (5)	2(10)	ns
Oral allergy syndrome	7 (35)	7 (35)	ns
PPIs at baseline, *n* (%)	7 (35)	9 (45)	ns
Prior use of topical steroids, *n* (%)	10 (50)	8 (40)	ns
Esophageal stricture dilation, *n* (%)	1 (5)	2 (10)	ns
Previous endoscopic intervention with food bolus extraction, *n* (%)	7 (35)	10 (50)	ns
Diagnostic delay,^a^ median (IQR)	4.5 (1–7.5)	3 (1–9.75)	ns
BMI (kg/m^2^), median (IQR)	24.1 (22.4–28.4)	24.0 (22.3–26.7)	ns
Nutrient intake			
Calcium (mg)	9.640	15.640	**.043** *
Folate (mg)	9.730	15.570	**.049** *
Food group intake			
Candy/sweets (g)	15.140	10.270	**.047** *
Low‐fat margarine (g)	15.540	9.770	**.045** *

*Note*: Bold values demonstrate significant values of *p* < .05.

Abbreviations: BMI, body mass index; EoE, eosinophilic esophagitis; FFED, four‐food elimination diet; FFED + AAF, four‐food elimination diet with addition of amino acid‐based formula; IQR, interquartile range; PPIs, proton pump inhibitors.

^a^ Time interval between first reported EoE symptoms and year of diagnosis.

* *p *< .05.

There were no statistically significant differences between the FFED and FFED + AAF groups at baseline, except for the intake of calcium, folate, candy/sweets, and low‐fat margarine.^36^ Overall, adherence to the dietary intervention was high and did not differ between the groups.^36^

### 3.2 PEC and nutritional intake analysis

The relationships between the intake of nutrients and food groups and PEC are presented in Table 2. Results for *p *< .1 are shown. All presented data are corrected for energy intake.

**Table 2:** Multiple multivariate regression analyses of the relationships between nutrients and food groups (per 1000 kCal), and PEC, corrected for age, gender, amino acid‐based formula use (yes/no) (only after 6 weeks), and baseline PEC (only after 6 weeks).

	Standardized beta (SE)	*p* Value	Adjusted explained variance	Nature of relationship
Relationship between nutrients per 1000 kCal intake and PEC *at baseline* (*n* = 39)				
Total carbohydrates (g)	0.389 (0.160)	**.013**	0.214	Unfavorable
Total protein (g)	−0.416 (0.160)	**.006**	0.245	Favorable
Total fat (g)	−0.357 (0.160)	**.026**	0.185	Favorable
Saturated fatty acids (g)	−0.305 (0.160)	.067	0.146	Favorable
Polyunsaturated fatty acids (g)	−0.274 (0.160)	.087	0.136	Favorable
Calcium (mg)	−0.327 (0.160)	.058	0.152	Favorable
Phosphorus (mg)	−0.377 (0.160)	**.013**	0.213	Favorable
Zinc (mg)	−0.395 (0.160)	**.009**	0.228	Favorable
Vitamin B12 (mg)	−0.319 (0.160)	**.039**	0.168	Favorable
Folate (mg)	−0.360 (0.160)	**.018**	0.199	Favorable
Relationship between nutrients per 1000 kCal intake and PEC *at 6 weeks* (*n* = 35)				
Vitamin C (mg)	−0.282 (0.169)	.066	0.321	Favorable
Relationship between foods/food groups per 1000 kCal intake and PEC *at baseline* (*n* = 39)				
Processed fruit juice/vegetable juice (g) (*n* = 14)	0.431 (0.160)	**.005**	0.254	Unfavorable
Beer (g) (*n* = 14)	0.273 (0.160)	.093	0.133	Unfavorable
White bread (g) (*n* = 21)	0.326 (0.160)	**.035**	0.173	Unfavorable
Ultra‐processed meals (ready‐to‐eat) (g) (*n* = 11)	0.302 (0.160)	.059	0.151	Unfavorable
Full‐fat cheese, and semiskimmed and full‐fat curd (g) (*n* = 22)	−0.331 (0.160)	.056	0.154	Favorable
Milk products (g) (*n* = 10)	−0.341 (0.160)	**.031**	0.178	Favorable
Relationship between foods/food groups and PEC *at 6 weeks* (*n* = 35)				
Food groups per 1000 kCal intake				
Coconut yogurt (g) (*n* = 10)	0.296 (0.169)	.087	0.310	Unfavorable
Hummus/legumes (g) (*n* = 20)	0.432 (0.169)	**.003**	0.441	Unfavorable
Peanuts (g) (*n* = 10)	0.293 (0.169)	.058	0.326	Unfavorable
Coffee/tea (g) (*n* = 32)	−0.316 (0.169)	**.048**	0.333	Favorable

*Note*: Bold values demonstrate significant values of *p *< .05, all values are presented for *p *< .1.

Abbreviation: PEC, peak eosinophil count/high power field.

#### 3.2.1 Relationship between nutrient intake and PEC at baseline

The intake of total protein (*β* = −.416; *p* = .006), total fat (*β* = −.357; *p* = .026), phosphorus (*β* = −.377; *p* = .013), zinc (*β* = −.395; *p* = .009), vitamin B12 (*β* = −.319; *p* = .039), and folate (*β* = −.360; *p* = .018) showed a negative (and thus favorable) statistically significant relationship with PEC. In contrast, the intake of total carbohydrates (*β* = .389; *p* = .013) showed a positive (and thus unfavorable) statistically significant relationship with PEC.

#### 3.2.2 Relationship between intake of foods and food groups and PEC at baseline

The intake of milk products (*β* = −.341; *p* = .031) showed a negative (and thus favorable) statistically significant relationship with PEC, whereas the intake of prepacked fruit juice/vegetable juice (*β* = .431; *p* = .005) as well as the intake of white bread (*β* = .326; *p* = .035) showed a positive (and thus unfavorable) statistically significant relationship with PEC.

#### 3.2.3 Relationship between nutrient intake and PEC after 6 weeks of dietary intervention

None of the nutrients showed a statistically significant relationship with PEC after the 6‐weeks intervention period.

#### 3.2.4 Relationship between intake of foods and food groups and PEC after 6 weeks of dietary intervention

The intake of coffee/tea (*β* = −.316; *p* = .048) showed a negative (and thus favorable) statistically significant relationship with PEC. The intake of hummus/legumes (*β* = .432; *p* = .003) revealed a positive (and thus unfavorable) statistically significant relationship with PEC.

## 4 DISCUSSION

In this prospective study, we evaluated associations between the nutritional intake of patients with EoE and disease severity to gain more insights into the possible aggregating and protecting effects of nutrients and food groups involved in EoE. Our findings suggest that nutritional components may either favorably or unfavorably impact the inflammation of the esophagus and thus the pathophysiology of EoE. This is an important finding as increased inflammation leads to damage of the epithelial surface, which lies at the origin of various manifestations of allergic diseases.^43^

Food sources rich in nutrients for which we found a favorable relationship with PEC are found in both animal and plant foods. Protein, fat, phosphorus, zinc, and vitamin B12 are found in meat, fish, dairy, and cheese (i.e., animal foods), while also several plant foods are rich in protein (e.g., nuts, legumes, soy), phosphorus (e.g., potatoes), and zinc (e.g., whole grains, nuts). The nutrients for which we found a *trend* for a favorable relationship with PEC, that is, saturated fat, LCPUFAs, calcium, and vitamin C are mostly found in meat, processed foods and full‐fat dairy and cheese (saturated fat, calcium), fatty fish, nuts and seeds, oil and margarines (LCPUFAs), and fruits and vegetables (vitamin C).

The favorable effect of total fat and saturated fat seems unexpected. However, the relevance of nutrients should be interpreted in light of foods consumed, thus by the source of the nutrients. The significant favorable effect of total protein, (saturated) fat, phosphorus, vitamin B12, zinc, and calcium could be partly explained by the intake of milk products, full‐fat cheese and semi‐skimmed or full‐fat curd, as these foods were related to lower inflammation. There is increasing evidence that moderate amounts of saturated fats from dairy products, in contrast to fat from meat and trans fatty acids, have anti‐inflammatory effects. We hypothesize that, due to its anti‐inflammatory effects, the intake of dairy, specifically full‐fat cheese in contrast to low‐fat cheese, could have a protective effect on esophageal inflammation as long as cow's milk allergy in EoE has not yet fully developed. In addition, LCPUFAs, including omega‐3 fatty acids (in fatty fish, walnuts, canola oil), contributed to the total fat intake and these fatty acids are known for their anti‐inflammatory effects.^44^ Furthermore, in contrast to skimmed dairy, full‐fat dairy is a source of vitamins A and D, and both are known for their immunomodulatory effects. Vitamin A is further acknowledged for its role in protective immune response mechanisms, since vitamin A is a key nutrient for mucosal integrity.^23^, ^45^ Moreover, fermented products, such as cheese, are increasingly recognized for their potential probiotic and postbiotic effects.^8^ Finally, cheese is a food product with a relatively high pH which could have a favorable effect in the esophagus.^46^, ^47^

The favorable effect of zinc on the disease severity of EoE can also be explained by its immunomodulatory effects. Zinc is suggested to exhibit a local effect on both the mucosal integrity, as well as on the mucosal cells of the gastrointestinal tract.^19^, ^20^, ^21^, ^22^, ^23^, ^24^, ^25^ Meat, dairy, and whole grains are rich sources of zinc.

The favorable effect of the intake of folate, and a trend for a favorable effect of vitamin C indicate that the intake of fruits and vegetables has beneficial effects, as fruits and vegetables are the major sources of folate and vitamin C.

These observations show similarities to the results of our previous study on habitual diet as related to the severity of EoE.^34^ In that study, we also found protective effects of dairy products, in particular fermented dairy, on the mucosal permeability of adult EoE patients.^34^ However, the favorable impact of protein, phosphorus, and vitamin B12 on inflammation in EoE in this study is in contrast to the unfavorable relationship of these nutrients with inflammation in EoE in our previous study by De Kroon et al.^34^ This could be explained by the fact that cheese intake in the study of De Kroon et al. was much lower (37 g/day), as compared to this study population, in which the intake of cheese products at baseline was twice as high (76 g/day).

The favorable relationship we found for coffee/tea might be explained by the high levels of polyphenols and flavonoids in coffee and (green) tea, associated with antioxidant, antiproliferative, and anti‐inflammation properties.^48^, ^49^

The observed unfavorable effect of the intake of total carbohydrates and white bread on the disease severity of EoE, can be explained by an unfavorable effect of mono‐ and disaccharides and refined grains. White bread consists of refined grains and is low in fiber. Fiber is known for its beneficial effects on the microbiome. These findings are in line with results of our previous study on habitual diet as related to the severity of EoE^34^ where we found a favorable relationship between dietary fiber intake and inflammation.

Another unfavorable effect was observed for processed food products, that is, processed fruit/vegetable juice and ultra‐processed meals, such as frozen pizza and ready‐to‐eat meals. Ultra‐processed foods have been consistently linked to other Western inflammatory diseases.^50^ Their adverse effects might be caused by their proinflammatory properties such as high levels of saturated or trans fatty acids, lack of dietary fiber, and low nutrient density,^48^ as well as by chemically modified ingredients, food additives, and ingredients produced under high pressure and extensive heat management methods. The presence of additives such as emulsifiers may negatively impact on the mucosal integrity,^51^ while highly heated foods may have high levels of advanced glycation end products which are suggested to have proinflammatory properties.^51^, ^52^, ^53^, ^54^ In addition, the unfavorable effect might be explained by the acidic effects of these food products. We hypothesize that the level of acidity of food products might influence the integrity of the esophagus mucosa, in which acidic foods might irritate the esophagus mucosa. Hence, products like soda and fruit juices have irritable effects due to their acidity, while, in contrast, basic foods with a higher pH such as cheese,^55^ could have a favorable effect. Finally, we hypothesize that the lack of microbial content of ultra‐processed foods is harmful, because these foods do not contribute to the total microbial exposure in the gut.

A remarkable finding was the unfavorable relationships of hummus (containing chickpeas and sometimes sesame), peanuts and dairy replacers based on coconut with PEC. Possibly, legumes, sesame, and peanut could be underestimated allergens for patients with EoE in the Netherlands. Coconut is high in (proinflammatory) saturated fatty acids,^40^ and showed a trend for an unfavorable effect on the disease severity of EoE.

Our findings do not implicate that the higher the intake of protein and fat the better, but they suggest that both animal foods, specifically (full‐fat and fermented) dairy, and plant foods in the right amounts may contribute beneficially to decreased inflammation. From many other studies, it is known that a healthy diet consists of the right proportions of plant and animal protein, (poly‐unsaturated) fat, and also carbohydrates rich in dietary fiber, predominantly provided by whole grains.^53^, ^56^

Overall, our results are in line with established anti‐ and proinflammatory effects of foods in other chronic inflammatory diseases as defined in the Dietary Inflammatory Index Development Study following an extensive literature review.^48^, ^57^

Strengths of the study were the accurate and standardized approach of obtaining patients' diet history and the reliable assessment of PEC using biopsies. In addition, this study further builds on our previous study^34^ on the relationship between patients' habitual diet and esophagus permeability and inflammation. These two studies are currently the only two studies looking at the effects of the habitual diet on EoE, making our study unique by highlighting immunomodulatory effects of the diet, other than elimination, and the link to the disease severity of EoE as a novel approach in the management and prevention of EoE.

This study has a few limitations. First, the relatively small sample size limits the reliability of the findings of our study. Second, the observational nature of our study design limits the ability to assign causality and may have led to residual confounding from unmeasured factors. Also, no correction for multiple testing was made as this study aimed to generate hypotheses. Third, the assessment of patients' nutritional status could be improved. We assessed BMI but did not determine vitamin and mineral status in patients. Malnutrition in chronic disease, such as allergic disease and Irritable Bowel Disease, promotes inflammation and vice versa,^58^, ^59^ in which iron is playing a key role.^60^ Malnutrition in EoE patients, specifically vitamin D deficiency, has been reported, although data are limited.^61^, ^62^ Also, the division of the food groups could be improved. For example, ready‐to‐eat and prepared meals were categorized as either Pancake and pizza or ready meals, and were not further divided into main ingredient or processing and preparation method. Also, the intake of herbs, spices, salt, and iodine (fortified in salt) was not reliably measured as we had not asked patients to weigh herbs, spices, and salt. Therefore, we could not reliably assess the intake of iodine as a significant amount of iodine intake in the Netherlands is supplied by the use of fortified salt. The intake of supplements was not assessed due to irregular intake. Fourth, in this study, no division was made for animal versus plant nutrients and food groups, while they have very distinctive effects, for instance, at the gut microbiome level.^28^, ^63^, ^64^ Furthermore, it is essential to note that some nutrients and food groups could have a synergistic, cumulative, or interactive effect on the disease severity of EoE.^57^ Therefore, in future studies, also food patterns should be studied. Finally, due to the known anti‐inflammatory effects of the AAF, we were not able to distinguish the nutritional effect from the intervention effect.

In conclusion, dietary compounds may play an important role in the pathology of EoE, in which dietary compounds have immunomodulatory effects, such as pro‐ or anti‐inflammatory influences. In addition to the elimination diet, a high‐quality and anti‐inflammatory composition of the diet of EoE patients is a promising adjuvant therapy for disease control during management and during the maintenance phase in EoE following disease remission to prevent relapse in EoE. In contrast, a proinflammatory diet could aggravate symptoms and decrease or even mask the management effects of elimination dietary therapy. Intervention trials with larger samples sizes that accurately quantify food intake and food patterns are needed for definite conclusions. Overall, better understanding of the possible aggravating or protecting effects of nutritional compounds is necessary for improved dietary advice and disease control in EoE.

## AUTHOR CONTRIBUTIONS

**Simone R. B. M. Eussen**: Conceptualization (supporting); methodology (supporting); software (lead); formal analysis (lead); writing—original draft (lead). **Sanne Wielders**: Software (supporting); formal analysis (supporting); writing—original draft (supporting); writing—review and editing (equal). **Willemijn E. de Rooij**: Writing—review and editing (equal). **Marleen T. J. Van Ampting**: Writing—review and editing (equal). **Betty C. A. M. Van Esch**: Conceptualization (supporting); writing—review and editing (equal). **Jeanne H. M. de Vries**: Writing—review and editing (equal). **Albert J. Bredenoord**: Conceptualization (supporting); writing—review and editing (equal). **Berber Vlieg‐Boerstra**: Conceptualization (lead); methodology (lead); writing—original draft (supporting); writing—review and editing (equal).

## CONFLICTS OF INTEREST STATEMENT

Simone R. B. M. Eussen, Marleen T. J. Van Ampting*, and Betty C. A. M. Van Esch are (former) employees of Danone Nutricia Research. Albert J. Bredenoord has received research funding from Nutricia, SST, Norgine, and Bayer; speaker and/or consulting fees from Laborie, Reckitt Benckiser, Robarts, EsoCap, Medtronic, DrFalk, Calypso, Regeneron, Celgene, AstraZeneca, and Arena and holds stocks SST. Berber Vlieg‐Boerstra received research funding from Nutricia, consulting or speaker's fees from Marfo Food groups, Nutricia, and Abbott. The remaining authors declare no conflict of interest.

## ETHICS STATEMENT

The study protocol was approved by the Medical Ethics Committee of Amsterdam University Medical Center, Amsterdam, the Netherlands. All participants provided written informed consent before taking part and were given a unique study ID to ensure anonymity.

## Supporting information

Supporting information.

Supporting information.

## Data Availability

The data that support the findings of this study are available on request from the corresponding author. The data are not publicly available due to privacy or ethical restrictions.

## References

[1] Dellon ES , Liacouras CA , Molina‐Infante J , et al. Updated international consensus diagnostic criteria for eosinophilic esophagitis: proceedings of the AGREE Conference. Gastroenterology. 2018;155(4):1022‐1033. 10.1053/j.gastro.2018.07.009 30009819 PMC6174113

[2] Warners MJ , Vlieg‐Boerstra BJ , Verheij J , et al. Elemental diet decreases inflammation and improves symptoms in adult eosinophilic oesophagitis patients. Aliment Pharmacol Ther. 2017;45(6):777‐787. 10.1111/apt.13953 28112427 PMC5324627

[3] de Rooij WE , Barendsen ME , Warners MJ , et al. Emerging incidence trends of eosinophilic esophagitis over 25 years: results of a nationwide register‐based pathology cohort. Neurogastroenterol Motil. 2021;33(7):e14072. 10.1111/nmo.14072 33426755 PMC8365671

[4] Dellon ES , Erichsen R , Baron JA , et al. The increasing incidence and prevalence of eosinophilic oesophagitis outpaces changes in endoscopic and biopsy practice: national population‐based estimates from Denmark. Aliment Pharmacol Ther. 2015;41(7):662‐670. 10.1111/apt.13129 25684441 PMC4504237

[5] Navarro P , Arias Á , Arias‐González L , Laserna‐Mendieta EJ , Ruiz‐Ponce M , Lucendo AJ . Systematic review with meta‐analysis: the growing incidence and prevalence of eosinophilic oesophagitis in children and adults in population‐based studies. Aliment Pharmacol Ther. 2019;49(9):1116‐1125. 10.1111/apt.15231 30887555

[6] Cianferoni A , Shuker M , Brown‐Whitehorn T , Hunter H , Venter C , Spergel JM . Food avoidance strategies in eosinophilic oesophagitis. Clin Exp Allergy. 2019;49(3):269‐284. 10.1111/cea.13360 30714219

[7] Dellon ES . Eosinophilic esophagitis: diagnostic tests and criteria. Curr Opin Gastroenterol. 2012;28(4):382‐388. 10.1097/MOG.0b013e328352b5ef 22450900 PMC4591255

[8] Gomez Torrijos E , Gonzalez‐Mendiola R , Alvarado M , et al. Eosinophilic esophagitis: review and update. Front Med. 2018;5:247. 10.3389/fmed.2018.00247 PMC6192373 30364207

[9] Kliewer KL , Cassin AM , Venter C . Dietary therapy for eosinophilic esophagitis: elimination and reintroduction. Clin Rev Allergy Immunol. 2018;55(1):70‐87. 10.1007/s12016-017-8660-1 29238902

[10] Gonsalves N . Steroids versus dietary therapy for the treatment of eosinophilic esophagitis. Curr Opin Gastroenterol. 2014;30(4):396‐401. 10.1097/mog.0000000000000086 24886760

[11] Kelly KJ , Lazenby AJ , Rowe PC , Yardley JH , Perman JA , Sampson HA . Eosinophilic esophagitis attributed to gastroesophageal reflux: improvement with an amino acid‐based formula. Gastroenterology. 1995;109(5):1503‐1512. 10.1016/0016-5085(95)90637-1 7557132

[12] Warners MJ , Vlieg‐Boerstra BJ , Bredenoord AJ . Elimination and elemental diet therapy in eosinophilic oesophagitis. Best Practice & Research Clinical Gastroenterology. 2015;29(5):793‐803. 10.1016/j.bpg.2015.06.013 26552778

[13] Reed CC , Fan C , Koutlas NT , Shaheen NJ , Dellon ES . Food elimination diets are effective for long‐term treatment of adults with eosinophilic oesophagitis. Aliment Pharmacol Ther. 2017;46(9):836‐844. 10.1111/apt.14290 28877359 PMC5659358

[14] Wang R , Hirano I , Doerfler B , Zalewski A , Gonsalves N , Taft T . Assessing adherence and barriers to long‐term elimination diet therapy in adults with eosinophilic esophagitis. Dig Dis Sci. 2018;63(7):1756‐1762. 10.1007/s10620-018-5045-0 29611076 PMC6166243

[15] 3rd Dawson DR, , Branch‐Mays G , Gonzalez OA , Ebersole JL . Dietary modulation of the inflammatory cascade. Periodontol 2000. 2014;64(1):161‐197. 10.1111/j.1600-0757.2012.00458.x 24320963

[16] European Food Safety Authority (EFSA) Panel on Dietetic Products Nutrition and Allergies . General scientific guidance for stakeholders on health claim applications. EFSA J. 2016;14(1):4367. 10.2903/j.efsa.2016.4367 PMC7996104 33791037

[17] European Food Safety Authority (EFSA) . Scientific opinion on the substantiation of health claims related to vitamin B6 and protein and glycogen metabolism (ID 65, 70, 71), function of the nervous system (ID 66), red blood cell formation (ID 67, 72, 186), function of the immune system (ID 68), regulation of hormonal activity (ID 69) and mental performance (ID 185) pursuant to article 13(1) of regulation (EC) no 1924/2006. EFSA J. 2009;7(9):1225. 10.2903/j.efsa.2009.1225

[18] Brown HA , Marnett LJ . Introduction to lipid biochemistry, metabolism, and signaling. Chem Rev. 2011;111(10):5817‐5820. 10.1021/cr200363s 21951202

[19] Brown CC , Noelle RJ . Seeing through the dark: new insights into the immune regulatory functions of vitamin A. Eur J Immunol. 2015;45(5):1287‐1295. 10.1002/eji.201344398 25808452 PMC4426035

[20] Cassani B , Villablanca EJ , De Calisto J , Wang S , Mora JR . Vitamin A and immune regulation: role of retinoic acid in gut‐associated dendritic cell education, immune protection and tolerance. Mol Aspects Med. 2012;33(1):63‐76. 10.1016/j.mam.2011.11.001 22120429 PMC3246074

[21] Dostal A , Lacroix C , Bircher L , et al. Iron modulates butyrate production by a child gut microbiota in vitro. mBio. 2015;6(6):e01453‐15. 10.1128/mBio.01453-15 26578675 PMC4659462

[22] Li Y , Hansen SL , Borst LB , Spears JW , Moeser AJ . Dietary iron deficiency and oversupplementation increase intestinal permeability, ion transport, and inflammation in pigs. J Nutr. 2016;146(8):1499‐1505. 10.3945/jn.116.231621 27358414 PMC4958291

[23] McCullough FSW , Northrop‐Clewes CA , Thurnham DI . The effect of vitamin A on epithelial integrity. Proc Nutr Soc. 1999;58(2):289‐293. 10.1017/s0029665199000403 10466169

[24] Miyoshi Y , Tanabe S , Suzuki T . Cellular zinc is required for intestinal epithelial barrier maintenance via the regulation of claudin‐3 and occludin expression. Am J Physiol Gastrointest Liver Physiol. 2016;311(1):G105‐G116. 10.1152/ajpgi.00405.2015 27151944

[25] Spencer SP , Belkaid Y . Dietary and commensal derived nutrients: shaping mucosal and systemic immunity. Curr Opin Immunol. 2012;24(4):379‐384. 10.1016/j.coi.2012.07.006 22857854 PMC3431603

[26] Neerven RJJ , Savelkoul H . Nutrition and allergic diseases. Nutrients. 2017;9(7):762. 10.3390/nu9070762 28714911 PMC5537876

[27] Marco ML , Heeney D , Binda S , et al. Health benefits of fermented foods: microbiota and beyond. Curr Opin Biotechnol. 2017;44:94‐102. 10.1016/j.copbio.2016.11.010 27998788

[28] Bolte LA , Vich Vila A , Imhann F , et al. Long‐term dietary patterns are associated with pro‐inflammatory and anti‐inflammatory features of the gut microbiome. Gut. 2021;70(7):1287‐1298. 10.1136/gutjnl-2020-322670 33811041 PMC8223641

[29] David LA , Maurice CF , Carmody RN , et al. Diet rapidly and reproducibly alters the human gut microbiome. Nature. 2014;505(7484):559‐563. 10.1038/nature12820 24336217 PMC3957428

[30] Moschen AR , Wieser V , Tilg H . Dietary factors: major regulators of the gut's microbiota. Gut Liver. 2012;6(4):411‐416. 10.5009/gnl.2012.6.4.411 23170142 PMC3493718

[31] Muir AB , Benitez AJ , Dods K , Spergel JM , Fillon SA . Microbiome and its impact on gastrointestinal atopy. Allergy. 2016;71(9):1256‐1263. 10.1111/all.12943 27240281 PMC4976690

[32] Wu GD , Chen J , Hoffmann C , et al. Linking long‐term dietary patterns with gut microbial enterotypes. Science. 2011;334(6052):105‐108. 10.1126/science.1208344 21885731 PMC3368382

[33] Hollingsworth JW , Maruoka S , Boon K , et al. In utero supplementation with methyl donors enhances allergic airway disease in mice. J Clin Invest. 2016;126(5):2012. 10.1172/jci87742 PMC4855943 27135881

[34] de Kroon MLA , Warners MJ , van Ampting MTJ , et al. The relationship of habitual diet with esophageal inflammation and integrity in eosinophilic esophagitis. Allergy. 2019;74(5):1005‐1009. 10.1111/all.13695 30515844

[35] de Kroon M , Eussen S , Holmes B , et al. The habitual diet of Dutch adult patients with eosinophilic esophagitis has pro‐inflammatory properties and low diet quality scores. Nutrients. 2021;13(1):214. 10.3390/nu13010214 33451130 PMC7828600

[36] de Rooij WE , Vlieg – Boerstra B , Warners MJ , et al. Effect of amino acid‐based formula added to four‐food elimination in adult eosinophilic esophagitis patients: a randomized clinical trial. Neurogastroenterol Motil. 2022;34(7):e14291. 10.1111/nmo.14291 34792264 PMC9286809

[37] de Rooij WE , Dellon ES , Parker CE , et al. Pharmacotherapies for the treatment of eosinophilic esophagitis: state of the art review. Drugs. 2019;79(13):1419‐1434. 10.1007/s40265-019-01173-2 31352605

[38] Westenbrink S , Jansen‐van der Vliet M. NEVO‐online 2016: achtergrondinformatie Bilthoven, the Netherlands: National Institute for Public Health and the Enviroment. 2016. NEVO‐online: achtergrondinformatie.

[39] Wageningen University and Research . 2023. Compl‐eat. https://compleat.nl/

[40] National Institute for Public Health and the Environment . NEVO online. Dutch Food Composition Database. http://nevo-online.rivm.nl/

[41] Brink L , Postma , Smeets A , Stafleu A , et al. Food‐based dietary guidelines for the Netherlands (Richtlijnen Schijf van Vijf) Den Haag, the Netherlands Stichting Voedingscentrum. 2020.

[42] van Rossum CTM , Fransen HP , Verkaik‐Kloosterman J , et al. Dutch National Food Consumption Survey 2007–2010. Diet of Children and Adults Aged 7 to 69 years. National Institute for Public Health and the Environment; 2011.

[43] Akdis CA . Does the epithelial barrier hypothesis explain the increase in allergy, autoimmunity and other chronic conditions? Nat Rev Immunol. 2021;21(11):739‐751. 10.1038/s41577-021-00538-7 33846604

[44] Venter C , Meyer RW , Nwaru BI , et al. EAACI position paper: influence of dietary fatty acids on asthma, food allergy, and atopic dermatitis. Allergy. 2019;74(8):1429‐1444. 10.1111/all.13764 31032983

[45] de Medeiros P , Pinto D , de Almeida J , et al. Modulation of intestinal immune and barrier functions by vitamin A: implications for current understanding of malnutrition and enteric infections in children. Nutrients. 2018;10(9):1128. 10.3390/nu10091128 30134532 PMC6164597

[46] Cunha P , Moreira A , Moreira P , Delgado L . Dietary diversity and childhood asthma‐dietary acid load, an additional nutritional variable to consider. Allergy. 2020;75(9):2418‐2420. 10.1111/all.14296 32929730

[47] Venter C , Smith PK , O'Mahony L . Reply to: dietary diversity and childhood asthma ‐ dietary acid load, an additional nutritional variable to consider. Allergy. 2020;75(9):2423. 10.1111/all.14343 32929728

[48] Shivappa N , Steck SE , Hurley TG , Hussey JR , Hébert JR . Designing and developing a literature‐derived, population‐based dietary inflammatory index. Public Health Nutr. 2014;17(8):1689‐1696. 10.1017/s1368980013002115 23941862 PMC3925198

[49] Maiti S , Nazmeen A , Medda N , Patra R , Ghosh TK . Flavonoids green tea against oxidant stress and inflammation with related human diseases. Clin Nutr Exp. 2019;24:1‐14. 10.1016/j.yclnex.2018.12.004

[50] Monteiro CA , Cannon G , Moubarac JC , Levy RB , Louzada MLC , Jaime PC . The UN decade of nutrition, the NOVA food classification and the trouble with ultra‐processing. Public Health Nutr. 2018;21(1):5‐17. 10.1017/s1368980017000234 28322183 PMC10261019

[51] Zinöcker M , Lindseth I . The Western Diet‐Microbiome‐Host interaction and its role in metabolic disease. Nutrients. 2018;10(3):365. 10.3390/nu10030365 29562591 PMC5872783

[52] Davis KE , Prasad C , Vijayagopal P , Juma S , Imrhan V . Advanced glycation end products, inflammation, and chronic metabolic diseases: links in a chain. Crit Rev Food Sci Nutr. 2016;56(6):989‐998. 10.1080/10408398.2012.744738 25259686

[53] Monteiro CA , Cannon G , Lawrence M , et al. Ultra‐processed foods, diet quality, and health using the NOVA classification system Rome, Italy Food and Agriculture Organization of the United Nations (FAO). 2019.

[54] Smith PK , Masilamani M , Li XM , Sampson HA . The false alarm hypothesis: food allergy is associated with high dietary advanced glycation end‐products and proglycating dietary sugars that mimic alarmins. J Allergy Clin Immunol. 2017;139(2):429‐437. 10.1016/j.jaci.2016.05.040 27544741

[55] Plessas S , Bosnea L , Alexopoulos A , Bezirtzoglou E . Potential effects of probiotics in cheese and yogurt production: a review. Eng Life Sci. 2012;12:433‐440. 10.1002/elsc.201100122

[56] Katz DL , Meller S . Can we say what diet is best for health. Annu Rev Public Health. 2014;35:83‐103. 10.1146/annurev-publhealth-032013-182351 24641555

[57] Vlieg‐Boerstra B , Groetch M , Vassilopoulou E , et al. The immune‐supportive diet inallergy management: a narrative review and proposal. Allergy. 2023;78(6):1441‐1458. 10.1111/all.15687 36802268

[58] Massironi S , Viganò C , Palermo A , et al. Inflammation and malnutrition in inflammatory bowel disease. Lancet Gastroenterol Hepatol. 2023;8(6):579‐590. 10.1016/s2468-1253(23)00011-0 36933563

[59] Roth‐Walter F , Berni Canani R , O'Mahony L , et al. Nutrition in chronic inflammatory conditions: bypassing the mucosal block for micronutrients. Allergy. 2024;79(2):353‐383. 10.1111/all.15972 38084827

[60] Roth‐Walter F . Iron‐Deficiency in atopic diseases: innate immune priming by allergens and siderophores. Front Allergy. 2022;3:859922. 10.3389/falgy.2022.859922 35769558 PMC9234869

[61] Slack MA , Ogbogu PU , Phillips G , Platts‐Mills TAE , Erwin EA . Serum vitamin D levels in a cohort of adult and pediatric patients with eosinophilic esophagitis. Ann Allergy Asthma Immunol. 2015;115(1):45‐50. 10.1016/j.anai.2015.04.016 26004426 PMC5448287

[62] Votto M , De Filippo M , Olivero F , et al. Malnutrition in eosinophilic gastrointestinal disorders. Nutrients. 2020;13(1):128. 10.3390/nu13010128 33396413 PMC7824578

[63] Rinninella E , Cintoni M , Raoul P , et al. Food components and dietary habits: keys for a healthy gut microbiota composition. Nutrients. 2019;11(10):2393. 10.3390/nu11102393 31591348 PMC6835969

[64] Singh RK , Chang HW , Yan D , et al. Influence of diet on the gut microbiome and implications for human health. J Transl Med 2017;15(1):73. 10.1186/s12967-017-1175-y. 28388917 PMC5385025

